# Bis[μ-*N*-(diethyl­amino-κ*N*)dimethyl­silylanilido-κ^2^ *N*:*N*]bis­[chlorido­cobalt(II)]

**DOI:** 10.1107/S1600536811054602

**Published:** 2011-12-23

**Authors:** Juan Chen

**Affiliations:** a Department of Chemistry, Taiyuan Teachers College, Taiyuan 030031, People’s Republic of China

## Abstract

In the title binuclear Co^II^ complex, [Co_2_(C_12_H_21_N_2_Si)_2_Cl_2_], an inversion center is located at the mid-point between the two Co atoms in the dimeric mol­ecule. The bidentate *N*-silylated anilide ligand coordinates the Co^II^ atom in an *N*,*N*′-chelating mode and provides the anilide N atom as a bridge to link two Co^II^ atoms. The two ends of the N—Si—N chelating unit exhibit different affinities for the metal atom. The Co—N_anilide_ bond is 2.031 (6) Å and Co—N_amino_ bond is 2.214 (6) Å. The four-coordinate Co atom presents a distorted tetra­hedral geometry, while the dimeric aggregation exhibits a (CoN)_2_ rhombus core with 1.998 (6) Å as the shortest sides and shows a ladder structure composed of Co, N and Si atoms.

## Related literature

For related reviews of metal amides, see: Holm *et al.* (1996 ▶); Kempe (2000 ▶). For catalytic applications of related *N*-silylated analido group 4 metal compounds towards olefin polymerization, see: Gibson *et al.* (1998 ▶); Hill & Hitchcock (2002 ▶); Yuan *et al.* (2010 ▶). For related organometallic compounds supported by analogous analido ligands, see: Schumann *et al.* (2000 ▶); Chen (2008 ▶, 2009 ▶). For related cobalt amides, see: Murray & Power (1984 ▶); Hope *et al.* (1985 ▶).
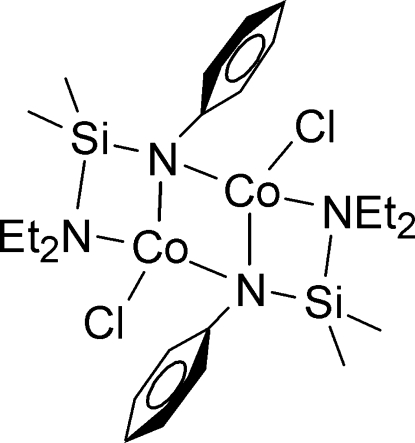


## Experimental

### 

#### Crystal data

[Co_2_(C_12_H_21_N_2_Si)_2_Cl_2_]
*M* *_r_* = 631.56
Orthorhombic, 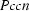

*a* = 12.180 (1) Å
*b* = 15.6753 (13) Å
*c* = 16.0235 (13) Å
*V* = 3059.3 (4) Å^3^
*Z* = 4
Mo *K*α radiation
μ = 1.36 mm^−1^
*T* = 293 K
0.20 × 0.15 × 0.10 mm

#### Data collection

Bruker SMART area-detector diffractometer
Absorption correction: multi-scan (*SADABS*; Sheldrick, 1996 ▶) *T*_min_ = 0.773, *T*_max_ = 0.876
16361 measured reflections
2885 independent reflections
1795 reflections with *I* > 2σ(*I*)
*R*_int_ = 0.093

#### Refinement

*R*[*F* ^2^ > 2σ(*F* ^2^)] = 0.072
*wR*(*F* ^2^) = 0.227
*S* = 1.17
2885 reflections
154 parameters
48 restraints
H-atom parameters constrained
Δρ_max_ = 1.97 e Å^−3^
Δρ_min_ = −0.51 e Å^−3^

### 

Data collection: *SMART* (Bruker, 2000 ▶); cell refinement: *SAINT* (Bruker, 2000 ▶); data reduction: *SAINT*; program(s) used to solve structure: *SHELXS97* (Sheldrick, 2008 ▶); program(s) used to refine structure: *SHELXL97* (Sheldrick, 2008 ▶); molecular graphics: *SHELXTL/PC* (Sheldrick, 2008 ▶); software used to prepare material for publication: *SHELXL97*.

## Supplementary Material

Crystal structure: contains datablock(s) I, global. DOI: 10.1107/S1600536811054602/jj2112sup1.cif

Structure factors: contains datablock(s) I. DOI: 10.1107/S1600536811054602/jj2112Isup2.hkl

Additional supplementary materials:  crystallographic information; 3D view; checkCIF report

## Figures and Tables

**Fig. 1.**
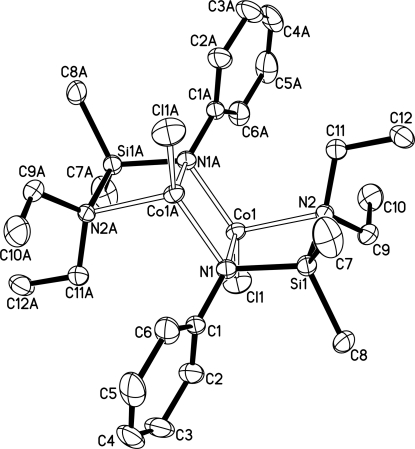
The molecular structure of (I), showing the atom-numbering scheme. Displacement ellipsoids are drawn at the 30% probability level. An inversion center is located at the mid-point between two Co atoms in the dimeric molecule. H atoms have been omitted for clarity.

## supplementary crystallographic information

### Comment

Metal amides are important substitutes for cyclopentadienyl derivatives and are found to have valuable applications in various industrial and biological processes (Holm *et al.*, 1996; Kempe, 2000). Group 4 metal amides supported with *N*-silylated anilido ligands are active catalysts for olefin polymerization reactions (Gibson *et al.*, 1998; Hill & Hitchcock, 2002). Recently, a class of monoionic *N*-silylated anilido ligands bearing a pendant amino group were the subject of focus presuming that the empty *d*-orbitals on silicon would interact with the lone-pair electrons on the *p*-orbital of nitrogen center through a *d—pπ* interaction throughout the N—Si—N motif.  Analogous compounds with different metals including Zn (Schumann *et al.*, 2000), Zr (Chen, 2009) and Fe (Chen, 2008) have been synthesized. In addition, a group of zirconium amides with a similar ligand were reported showing good performance in ethylene polymerization reactions (Yuan *et al.*, 2010). In view of the importance of these compounds, the synthesis and crystal structure of a new cobalt(II) anilido complex, (I), is reported.

The title compound, [Co(C_12_H_21_N_2_Si)Cl]_2_, is a binuclear Co^II^ complex with an inversion center located at the mid-point between two Co atoms in the dimeric molecule (Fig. 1). Each Co(II) atom is bound to three nitrogen atoms and one chlorine atom, resulting in a distorted tetrahedral geomety at the metal center.  The bidentate *N*-silylated anilide ligand coordinates a metal center in an *N*,*N'*-chelating mode and provides the anilido nitrogen as a bridge to link the two Co atoms. The two ends of the N—Si—N chelating unit exhibit different affinities for the metal center. The Co—Co distance is 2.5682 (19) Å, which is similar to 2.583 (1) Å in [Co{N(SiMe_3_)_2_}_2_]_2_ and 2.566 (3) Å in [Co(NPh_2_)_2_]_2_ (Murray & Power, 1984; Hope *et al.*, 1985).  The N—Si—N angle is constrained to be 98.8 (3)°.  The Co—N_anilido_ bond is 2.031 (2) Å and Co—N_amino_ bond is 2.214 (6) Å.  The four-coordinate Co atom presents a distorted tetrahedral geometry while the dimeric aggregation exhibits a (CoN)_2_ rhombus core with 2.0 Å sides and shows a ladder structure composed of Co, N and Si atoms.

### Experimental

The title compound was prepared by a one-pot reaction of LiBu*^n^*, *N*-[(diethylamino)dimethylsilyl]aniline and CoCl_2_ as follows: A solution of LiBu*^n^* (1.6 *M*, 1.75 ml, 2.8 mmol) in hexane was slowly added into a solution of *N*-[(diethylamino)dimethylsilyl]aniline (0.62 g, 2.8 mmol) in Et_2_O (20 ml) at 273 K by syringe. The mixture was stirred at room temperature for two hours and then added to a stirring suspension of CoCl_2_ (0.37 g, 2.8 mmol) in Et_2_O (20 ml) at 273 K. The resulting mixture was stirred at room temperature for 8 h. Then all the volatiles were removed under vacuum. The residue was extracted with toluene (25 ml). The filtrate was concentrated and suitable green single-crystals of the title compound were obtained by recrystallization in toluene. (yield 0.25 g, 28%). Anal. Calc. for C_24_H_42_Cl_2_Co_2_N_4_Si_2_: C, 45.64; H, 6.70; N, 8.87%.  Found: C, 45.48; H, 6.65; N, 9.05%.

### Refinement

All of the H atoms were placed in their geometrically idealized positions and constrained to ride on their parent atoms with calculated positions and then refined using the riding model with Atom—H lengths of 0.93Å (CH), 0.97 Å (CH_2_) or 0.96Å (CH_3_). Isotropic displacement parameters for these atoms were set to 1.2 (CH, CH_2_) or 1.5 (CH_3_) times *U*_eq_ of the parent atom. The N—Si—N angle is constrained to be 98.8 (3)°.

### Crystal data

**Table:**

[Co_2_(C_12_H_21_N_2_Si)_2_Cl_2_]	*F*(000) = 1320
*M**_r_* = 631.56	*D*_x_ = 1.371 Mg m^−^^3^
Orthorhombic, *P**c**c**n*	Mo *K*α radiation, λ = 0.71073 Å
Hall symbol: -P 2ab 2ac	Cell parameters from 3365 reflections
*a* = 12.180 (1) Å	θ = 2.3–26.3°
*b* = 15.6753 (13) Å	µ = 1.36 mm^−^^1^
*c* = 16.0235 (13) Å	*T* = 293 K
*V* = 3059.3 (4) Å^3^	Block, green
*Z* = 4	0.20 × 0.15 × 0.10 mm

### Data collection

**Table:**

Bruker SMART area-detector diffractometer	2885 independent reflections
Radiation source: fine-focus sealed tube	1795 reflections with *I* > 2σ(*I*)
graphite	*R*_int_ = 0.093
Detector resolution: 7.9 pixels mm^-1^	θ_max_ = 25.6°, θ_min_ = 2.1°
φ and ω scan	*h* = −14→14
Absorption correction: multi-scan (*SADABS*; Sheldrick, 1996)	*k* = −17→19
*T*_min_ = 0.773, *T*_max_ = 0.876	*l* = −13→19
16361 measured reflections	

### Refinement

**Table:**

Refinement on *F*^2^	Primary atom site location: structure-invariant direct methods
Least-squares matrix: full	Secondary atom site location: difference Fourier map
*R*[*F*^2^ > 2σ(*F*^2^)] = 0.072	Hydrogen site location: inferred from neighbouring sites
*wR*(*F*^2^) = 0.227	H-atom parameters constrained
*S* = 1.17	*w* = 1/[σ^2^(*F*_o_^2^) + (0.0945*P*)^2^ + 13.3754*P*] where *P* = (*F*_o_^2^ + 2*F*_c_^2^)/3
2885 reflections	(Δ/σ)_max_ < 0.001
154 parameters	Δρ_max_ = 1.97 e Å^−^^3^
48 restraints	Δρ_min_ = −0.51 e Å^−^^3^

### Special details

**Table:**

Experimental. MS (EI, 70 eV): *m/z* 632 [*M*]^+^.
Geometry. All e.s.d.'s (except the e.s.d. in the dihedral angle between two l.s. planes) are estimated using the full covariance matrix. The cell e.s.d.'s are taken into account individually in the estimation of e.s.d.'s in distances, angles and torsion angles; correlations between e.s.d.'s in cell parameters are only used when they are defined by crystal symmetry. An approximate (isotropic) treatment of cell e.s.d.'s is used for estimating e.s.d.'s involving l.s. planes.
Refinement. Refinement of *F*^2^ against ALL reflections. The weighted *R*-factor *wR* and goodness of fit *S* are based on *F*^2^, conventional *R*-factors *R* are based on *F*, with *F* set to zero for negative *F*^2^. The threshold expression of *F*^2^ > σ(*F*^2^) is used only for calculating *R*-factors(gt) *etc*. and is not relevant to the choice of reflections for refinement. *R*-factors based on *F*^2^ are statistically about twice as large as those based on *F*, and *R*- factors based on ALL data will be even larger.

### Fractional atomic coordinates and isotropic or equivalent isotropic displacement parameters (Å^2^)

**Table:**

	*x*	*y*	*z*	*U*_iso_*/*U*_eq_	
Co1	0.55911 (8)	0.48017 (6)	0.43654 (6)	0.0307 (3)	
Si1	0.37762 (17)	0.55954 (13)	0.36970 (13)	0.0356 (5)	
Cl1	0.71974 (18)	0.48946 (17)	0.37397 (16)	0.0617 (7)	
N1	0.4612 (5)	0.5831 (4)	0.4563 (4)	0.0328 (14)	
N2	0.4195 (5)	0.4511 (4)	0.3528 (4)	0.0303 (13)	
C1	0.4863 (7)	0.6714 (5)	0.4684 (6)	0.0472 (14)	
C2	0.5770 (8)	0.7089 (5)	0.4330 (6)	0.0534 (14)	
H2A	0.6230	0.6770	0.3988	0.064*	
C3	0.6006 (9)	0.7956 (6)	0.4482 (6)	0.0601 (15)	
H3A	0.6627	0.8207	0.4250	0.072*	
C4	0.5322 (9)	0.8417 (6)	0.4969 (6)	0.0620 (15)	
H4A	0.5474	0.8991	0.5059	0.074*	
C5	0.4443 (8)	0.8075 (6)	0.5320 (7)	0.0607 (15)	
H5A	0.3997	0.8408	0.5660	0.073*	
C6	0.4175 (8)	0.7207 (5)	0.5182 (6)	0.0536 (14)	
H6A	0.3549	0.6971	0.5420	0.064*	
C7	0.2306 (8)	0.5722 (7)	0.3939 (8)	0.079 (4)	
H7A	0.1963	0.6064	0.3516	0.119*	
H7B	0.2226	0.5996	0.4472	0.119*	
H7C	0.1962	0.5171	0.3957	0.119*	
C8	0.4136 (10)	0.6277 (6)	0.2788 (6)	0.073 (3)	
H8A	0.3484	0.6541	0.2573	0.109*	
H8B	0.4465	0.5932	0.2361	0.109*	
H8C	0.4646	0.6710	0.2960	0.109*	
C9	0.4621 (7)	0.4311 (5)	0.2680 (5)	0.0456 (18)	
H9A	0.5153	0.4742	0.2523	0.055*	
H9B	0.4019	0.4338	0.2285	0.055*	
C10	0.5154 (8)	0.3447 (6)	0.2621 (6)	0.057 (2)	
H10A	0.5407	0.3355	0.2060	0.085*	
H10B	0.4629	0.3014	0.2765	0.085*	
H10C	0.5765	0.3420	0.2998	0.085*	
C11	0.3406 (7)	0.3856 (5)	0.3839 (5)	0.0468 (19)	
H11A	0.3119	0.4042	0.4374	0.056*	
H11B	0.3800	0.3326	0.3930	0.056*	
C12	0.2441 (8)	0.3683 (6)	0.3251 (6)	0.056 (2)	
H12A	0.1971	0.3257	0.3492	0.083*	
H12B	0.2714	0.3483	0.2724	0.083*	
H12C	0.2032	0.4200	0.3169	0.083*	

### Atomic displacement parameters (Å^2^)

**Table:**

	*U*^11^	*U*^22^	*U*^33^	*U*^12^	*U*^13^	*U*^23^
Co1	0.0344 (5)	0.0272 (5)	0.0305 (6)	0.0036 (4)	0.0043 (4)	0.0027 (4)
Si1	0.0450 (12)	0.0250 (11)	0.0369 (12)	0.0043 (9)	−0.0108 (10)	0.0007 (9)
Cl1	0.0470 (12)	0.0727 (17)	0.0655 (15)	0.0029 (11)	0.0246 (11)	0.0051 (12)
N1	0.040 (3)	0.023 (3)	0.036 (4)	0.005 (2)	−0.006 (3)	0.001 (2)
N2	0.045 (3)	0.018 (3)	0.028 (3)	0.000 (2)	−0.004 (3)	−0.002 (2)
C1	0.064 (3)	0.024 (3)	0.053 (3)	0.001 (2)	−0.020 (3)	0.003 (2)
C2	0.069 (3)	0.030 (3)	0.061 (3)	−0.004 (2)	−0.019 (3)	0.006 (2)
C3	0.077 (3)	0.035 (3)	0.068 (3)	−0.006 (3)	−0.022 (3)	0.007 (3)
C4	0.082 (3)	0.035 (3)	0.069 (3)	−0.001 (3)	−0.025 (3)	0.002 (2)
C5	0.081 (3)	0.036 (3)	0.066 (3)	0.007 (3)	−0.022 (3)	−0.003 (2)
C6	0.072 (3)	0.030 (3)	0.059 (3)	0.005 (2)	−0.020 (3)	−0.001 (2)
C7	0.058 (6)	0.079 (8)	0.100 (9)	0.027 (6)	−0.024 (6)	−0.027 (7)
C8	0.139 (10)	0.036 (5)	0.042 (5)	−0.014 (6)	−0.035 (6)	0.010 (4)
C9	0.060 (5)	0.043 (4)	0.034 (4)	−0.003 (4)	0.005 (3)	0.001 (3)
C10	0.074 (5)	0.051 (5)	0.046 (4)	0.004 (4)	0.007 (4)	−0.011 (4)
C11	0.056 (4)	0.036 (4)	0.048 (4)	−0.008 (3)	−0.012 (4)	0.006 (3)
C12	0.062 (4)	0.043 (4)	0.062 (5)	−0.014 (4)	−0.017 (4)	0.007 (4)

### Geometric parameters (Å, °)

**Table:**

Co1—N1^i^	1.998 (6)	C5—C6	1.416 (12)
Co1—N1	2.031 (6)	C5—H5A	0.9300
Co1—Cl1	2.203 (2)	C6—H6A	0.9300
Co1—N2	2.214 (6)	C7—H7A	0.9600
Co1—Co1^i^	2.5682 (19)	C7—H7B	0.9600
Co1—Si1	2.754 (2)	C7—H7C	0.9600
Si1—N1	1.760 (6)	C8—H8A	0.9600
Si1—N2	1.795 (6)	C8—H8B	0.9600
Si1—C7	1.843 (10)	C8—H8C	0.9600
Si1—C8	1.859 (9)	C9—C10	1.505 (12)
N1—C1	1.431 (9)	C9—H9A	0.9700
N1—Co1^i^	1.998 (6)	C9—H9B	0.9700
N2—C9	1.487 (10)	C10—H10A	0.9600
N2—C11	1.492 (10)	C10—H10B	0.9600
C1—C2	1.375 (13)	C10—H10C	0.9600
C1—C6	1.391 (13)	C11—C12	1.531 (11)
C2—C3	1.410 (12)	C11—H11A	0.9700
C2—H2A	0.9300	C11—H11B	0.9700
C3—C4	1.351 (14)	C12—H12A	0.9600
C3—H3A	0.9300	C12—H12B	0.9600
C4—C5	1.323 (14)	C12—H12C	0.9600
C4—H4A	0.9300		
			
N1^i^—Co1—N1	100.8 (2)	C5—C4—C3	121.9 (10)
N1^i^—Co1—Cl1	122.26 (18)	C5—C4—H4A	119.1
N1—Co1—Cl1	122.67 (19)	C3—C4—H4A	119.1
N1^i^—Co1—N2	108.9 (2)	C4—C5—C6	120.6 (10)
N1—Co1—N2	78.9 (2)	C4—C5—H5A	119.7
Cl1—Co1—N2	114.83 (18)	C6—C5—H5A	119.7
N1^i^—Co1—Co1^i^	50.96 (17)	C1—C6—C5	119.0 (9)
N1—Co1—Co1^i^	49.84 (17)	C1—C6—H6A	120.5
Cl1—Co1—Co1^i^	147.37 (10)	C5—C6—H6A	120.5
N2—Co1—Co1^i^	95.69 (16)	Si1—C7—H7A	109.5
N1^i^—Co1—Si1	117.33 (18)	Si1—C7—H7B	109.5
N1—Co1—Si1	39.67 (17)	H7A—C7—H7B	109.5
Cl1—Co1—Si1	120.40 (9)	Si1—C7—H7C	109.5
N2—Co1—Si1	40.57 (15)	H7A—C7—H7C	109.5
Co1^i^—Co1—Si1	75.44 (6)	H7B—C7—H7C	109.5
N1—Si1—N2	98.8 (3)	Si1—C8—H8A	109.5
N1—Si1—C7	111.9 (4)	Si1—C8—H8B	109.5
N2—Si1—C7	114.2 (4)	H8A—C8—H8B	109.5
N1—Si1—C8	111.2 (4)	Si1—C8—H8C	109.5
N2—Si1—C8	111.0 (4)	H8A—C8—H8C	109.5
C7—Si1—C8	109.4 (6)	H8B—C8—H8C	109.5
N1—Si1—Co1	47.44 (19)	N2—C9—C10	113.5 (7)
N2—Si1—Co1	53.33 (19)	N2—C9—H9A	108.9
C7—Si1—Co1	138.3 (4)	C10—C9—H9A	108.9
C8—Si1—Co1	112.0 (4)	N2—C9—H9B	108.9
C1—N1—Si1	115.7 (5)	C10—C9—H9B	108.9
C1—N1—Co1^i^	112.9 (5)	H9A—C9—H9B	107.7
Si1—N1—Co1^i^	120.1 (3)	C9—C10—H10A	109.5
C1—N1—Co1	131.6 (5)	C9—C10—H10B	109.5
Si1—N1—Co1	92.9 (3)	H10A—C10—H10B	109.5
Co1^i^—N1—Co1	79.2 (2)	C9—C10—H10C	109.5
C9—N2—C11	112.6 (6)	H10A—C10—H10C	109.5
C9—N2—Si1	115.9 (5)	H10B—C10—H10C	109.5
C11—N2—Si1	114.7 (5)	N2—C11—C12	114.2 (7)
C9—N2—Co1	109.2 (5)	N2—C11—H11A	108.7
C11—N2—Co1	115.7 (4)	C12—C11—H11A	108.7
Si1—N2—Co1	86.1 (2)	N2—C11—H11B	108.7
C2—C1—C6	118.9 (8)	C12—C11—H11B	108.7
C2—C1—N1	122.0 (8)	H11A—C11—H11B	107.6
C6—C1—N1	119.2 (8)	C11—C12—H12A	109.5
C1—C2—C3	120.4 (10)	C11—C12—H12B	109.5
C1—C2—H2A	119.8	H12A—C12—H12B	109.5
C3—C2—H2A	119.8	C11—C12—H12C	109.5
C4—C3—C2	119.3 (10)	H12A—C12—H12C	109.5
C4—C3—H3A	120.4	H12B—C12—H12C	109.5
C2—C3—H3A	120.4		

Symmetry codes: (i) −*x*+1, −*y*+1, −*z*+1.

## References

[bb1] Bruker (2000). *SMART* and *SAINT* Bruker AXS Inc., Madison, Wisconsin, USA.

[bb2] Chen, J. (2008). *Acta Cryst.* E**64**, m938. 10.1107/S1600536808018114 PMC2961661 21202791

[bb3] Chen, J. (2009). *Acta Cryst.* E**65**, m1307. 10.1107/S1600536809039804 PMC2970990 21578071

[bb4] Gibson, V. C., Kimberley, B. S., White, A. J. P., Williams, D. J. & Howard, P. (1998). *Chem. Commun.* pp. 313–314.

[bb5] Hill, M. S. & Hitchcock, P. B. (2002). *Organometallics*, **21**, 3258–3262.

[bb6] Holm, R. H., Kenneppohl, P. & Solomon, E. I. (1996). *Chem. Rev.* **96**, 2239–2314. 10.1021/cr9500390 11848828

[bb7] Hope, H., Olmstead, M. M., Murray, B. D. & Power, P. P. (1985). *J. Am. Chem. Soc.* **107**, 712–713.

[bb8] Kempe, R. (2000). *Angew. Chem. Int. Ed.* **39**, 468–493. 10.1002/(sici)1521-3773(20000204)39:3<468::aid-anie468>3.0.co;2-g 10671235

[bb9] Murray, B. D. & Power, P. P. (1984). *Inorg. Chem.* **23**, 4584–4588.

[bb10] Schumann, H., Gottfriedsen, J., Dechert, S. & Girgsdies, F. (2000). *Z. Anorg. Allg. Chem.* **626**, 747–758.

[bb11] Sheldrick, G. M. (1996). *SADABS* University of Göttingen, Germany.

[bb12] Sheldrick, G. M. (2008). *Acta Cryst.* A**64**, 112–122. 10.1107/S0108767307043930 18156677

[bb13] Yuan, S. F., Wei, X. H., Tong, H. B., Zhang, L. P., Liu, D. S. & Sun, W. H. (2010). *Organometallics*, **29**, 2085–2092.

